# Robust performance comparison of PMSM for flight control applications in more electric aircraft

**DOI:** 10.1371/journal.pone.0283541

**Published:** 2023-07-07

**Authors:** Djaloul Karboua, Toual Belgacem, Zeashan Hameed Khan, Cherif Kellal

**Affiliations:** 1 Applied Automation and Industrial Diagnostics Lab (LAADI), University of Djelfa, Djelfa, Algeria; 2 Department of Avionics Engineering, CAE, National University of Sciences & Technology (NUST), Islamabad, Pakistan; J.C. Bose University of Science and Technology, YMCA, INDIA, INDIA

## Abstract

This paper describes a robust performance comparison of flight control actuation controllers based on a permanent magnet synchronous motor (PMSM) in more electric aircraft (MEA). Recently, the PMSM has become a favorite for the flight control applications of more electric aircraft (MEA) due to their improved efficiency, higher torque, less noise, and higher reliability as compared to their counterparts. Thus, advanced nonlinear control techniques offer even better performance for the control of PMSM as noticed in this research. In this paper, three nonlinear approaches i.e. Feedback Linearization Control (FBL) through the cancellation of the non-linearity of the system, the stabilization of the system via Backstepping Control (BSC) using the Lyapunov candidate function as well as the robust performance with chattering minimization by applying the continuous approximation based Sliding Mode Control (SMC) are compared with generalized Field-Oriented Controller (FOC). The comparison of FOC, FBL, BSC and SMC shows that the nonlinear controllers perform well under varying aerodynamic loads during flight. However, the performance of the sliding mode control is found superior as compared to the other three controllers in terms of better performance characteristics e.g. response time, steady-state error etc. as well as the control robustness in the presence of the uncertain parameters of the PMSM model and variable load torque acting as a disturbance. In essence, the peak of the tolerance band is less than 20% for all nonlinear and FOC controller, while it is less than 5% for SMC. Steady state error for the SMC is least (0.01%) as compared to other three controllers. Moreover, the SMC controller is able to withstand 50% parameter variation and loading torque of 10 N.m without significant changes in performance. Six simulation scenarios are used to analyze the performance and robustness which depict that the sliding mode controller performs well in terms of the desired performance for MEA application.

## Introduction

In traditional commercial aircraft technology, all non-propulsive systems are driven by a combination of secondary power sources comprising of mechanical, electric, hydraulic and pneumatic systems which results in increased cost, weight and reduced reliability [1]. With the advent of recent innovation in electrical machine design and power electronics, reliable actuators for flight controls are replacing the conventional architecture resulting in more electric aircraft (MEA) e.g. Boeing 787, Airbus 380 and F-35 from Lockheed [2]. In future, the technology is being directed for an all-electric aircraft (AEA). Most significant gain due to this technology shift has resulted in lower fuel consumption, lower emissions and noise while reducing failure rate and offering higher dependability as compared to conventional technology [3]. However, this modification has increased the power demand on-board aircraft which has increased to 1 MW (Boeing-777) as compared to older designs which used only one-fourth of it [4].

**Fig 1:**
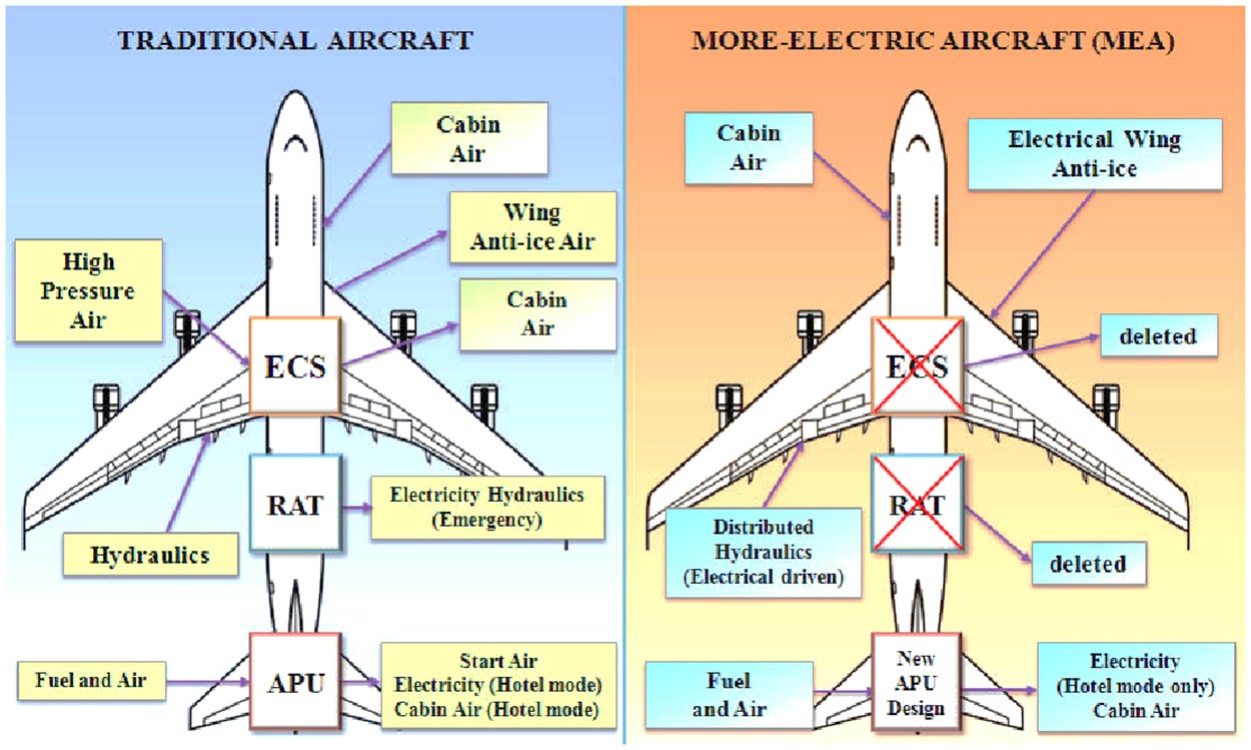
Comparison of conventional vs. more electric aircraft (MEA) technology [3].

**Fig 2:**
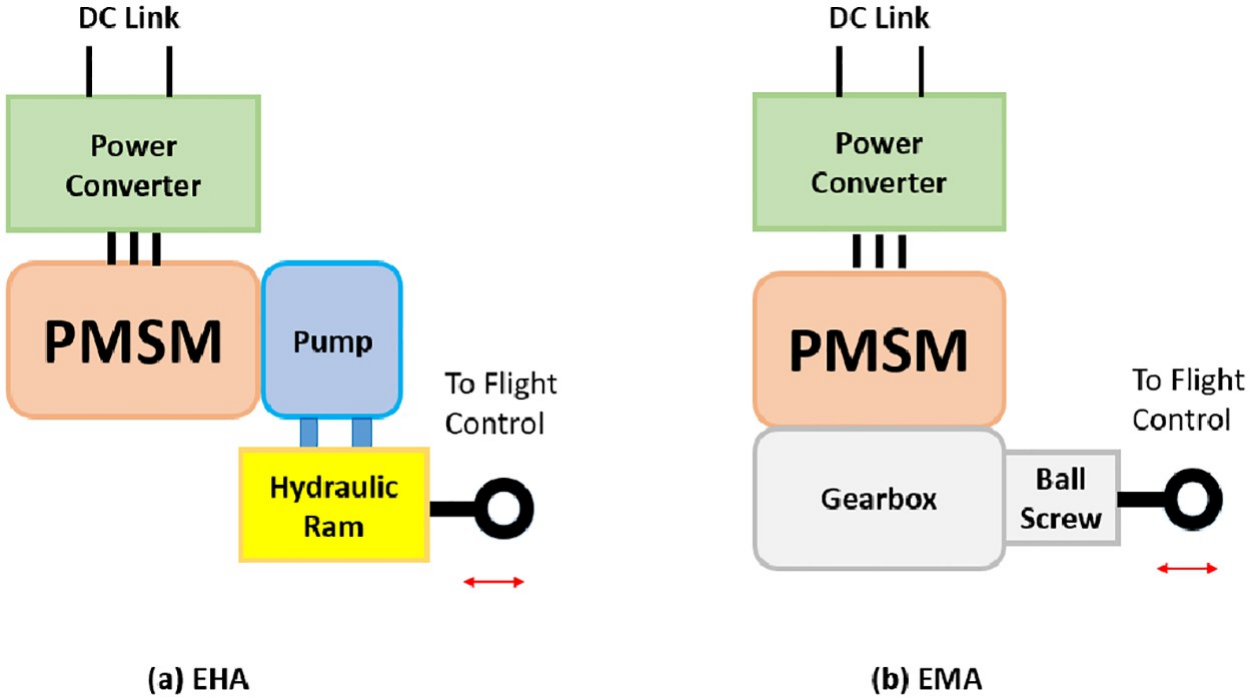
Flight control actuators in MEA. a) Electrohydrostatic b) Electromechanical Actuator.

Fig 1 compares the two architectures where it is clearly evident that the environmental control system (ECS) and Ram air turbine (RAT) are removed in the MEA technology while anti-icing system is electrically driven with modified auxiliary power unit (APU) design. The distributed flight control surfaces are hydraulic but electrically driven. Permanent magnet synchronous motors (PMSMs) are one of the most widely used machines in aerospace electromechanical or electrohydrostatic actuators (EMA/EHA) for MEA flight control applications [5]. The predominance of the permanent magnet synchronous motor is explained by its three great advantages: compactness, a high torque to current ratio and low maintenance cost [3]. The two actuating technologies are compared in Fig 2.

PMSM based electrical actuators are a popular choice due to their superior performance as represented by higher steady-state torque as compared to induction machines, simpler controller of the PM motor, high power density, and high efficiency because of reduced rotor losses. They have occupied a valuable place in applications such as industrial automation, robotics and electric vehicles (EVs). Despite the advantages of PMSM, it needs a well designed controller to reach its best performance. The world of motor control is classified into two categories; i.e. scalar control and vector control. The vector control is further classified into classical control and advanced control [6, 7]. The classical control usually comprises of a simple scheme such as a Proportional-Integral-Derivative (PID) regulator based Field-Oriented Control (FOC) and Direct Torque Control (DTC). However, these controllers are sensitive to parameter variation as well as changes in torque and speed during continuous operation. In order to overcome this problem, designers have implemented nonlinear control techniques [8–11]. Several advanced algorithms e.g. Backstepping (BSC), Sliding Mode (SMC), Feedback Linearization (FBLC), Passivity-Based (PBC) and *H*_∞_ control as well as optimal control techniques like Model Predictive (MPC), LQR, LQI and LQG, Adaptive controllers including direct or indirect MRAC, parameter variation control (PVC), Extended Method (EMC), observation and estimation approach, Intelligent control techniques e.g. Fuzzy Logic Control (FLC), ANFIS and other variants have been presented so far [12]. They are applied to PMSM system to achieve the desired drive performance and to achieve a robust dynamic response. However, sliding mode control appears to be one of the outstanding robust control technique for the actuation of MEA flight control applications due to its reliable performance while simultaneously offering higher robustness under uncertainty and external disturbance [13]. In addition, it is characterized by a simple structure and quick response in tracking control on the foreseen sliding surface [14–16].

Feedback linearization (FBL) approach transforms the nonlinear system dynamics into a linear system in order to simplify the design of controllers to ensure global stability. The vector based control technique based on feedback linearization is an innovative new control scheme [17]. Backstepping control is another design method for the feedback control of uncertain nonlinear system. The nonlinear model includes identification of the state variables, inputs, and outputs for the control design. Furthermore, it is based on a Lyapunov candidate function (CLF) to study the stabilization of the system [18, 19]. A comparison of robust control techniques for PMSM is recently reported in which H_∞_ robust control and sliding mode controller (SMC) are critically compared [20]. Likewise, research on the intelligent control of PMSM using fuzzy based multi-variable optimization approach is also reported [21]. However, no such comparison is so far made for nonlinear vector based controllers. In the present work, we are focusing on PMSM instead of the complete actuator design with an understanding that this is the key dynamic part of the actuation system which needs sufficient design consideration. Then, we proceed to obtain a performance comparison between three types of vector control methods i.e., the Field-Oriented Control (FOC), Feedback Linearization Control (FBL), Backstepping Control (BSC) and the Sliding Mode Control based continuous approximation method (SMC) are presented to gain an insight of the design process and clear understanding of the control performance for fail-safe aerospace applications as well as to achieve the desired characteristic performance and durability under the presence of uncertainties of the actuation drive parameters and external (or internal) plant disturbances. The remainder of this work is organized as follows: In Section 2, the mathematical modeling of the PMSM is presented. The design of three vector control schemes namely FOC, FBL, and BSC are described in Section 3. Section 4 presents the simulation results of the comparative performance of all four controllers by considering several scenarios to assess robustness. Finally, Section 5 concludes the paper.

## PMSM model

PMSM is a nonlinear multi-input multi-output (MIMO) dynamic system, where, the voltages are input, the state variables are direct and quadratic current, and the output is defined by speed and electromagnetic torque. The modeling of a PMSM can be done in several ways which consist of differential equations, transfer function or state-space method. The dynamic model of a PMSM is presented in Eqs (1) and (2) as under [22, 23]:

$$\begin{array}{c} \left\{ \begin{array}{cc} u_{d}=R_{s}.i_{d}+L_{d}.\frac{d}{dt}i_{d}-L_{q}.\omega_{e}.i_{q} & \;\\ u_{q}=R_{s}.i_{q}+L_{q}.\frac{d}{dt}i_{q}+L_{d}.\omega_{e}.i_{d}+\Phi_{f}.\omega_{e} & \; \end{array}\right. \end{array}$$ (1)

and

$$\begin{array}{c} \left\{ \begin{array}{cc} T_{e}=\frac{3.p}{2}((L_{d}-L_{q}).i_{d}.i_{q}+\Phi_{f}.i_{q}) & \;\\ J.\frac{d}{dt}\omega_{r}+F.\omega_{r}=T_{e}-T_{r} & \; \end{array}\right. \end{array}$$ (2)

where: *ω*_*e*_ = *p*.*ω*_*r*_, *i*_*d*_, *i*_*q*_ are d-q axis equivalent stator currents; *u*_*d*_, *u*_*q*_ are d-q axis equivalent stator voltages; *ω*_*r*_, *ω*_*e*_ are mechanical (rotor) and electrical (stator) speed respectively; *p* is the number of pole pairs; *R*_*s*_ is the stator resistance per phase; *L*_*d*_, *L*_*q*_ are d-q axis equivalent stator inductances; *T*_*e*_, *T*_*r*_ are electromagnetic and load torques; *J* is the moment of inertia of the rotor; *F* is the friction constant of the rotor and *Φ*_*f*_ is the rotor magnetic flux linking the stator.

**Fig 3:**
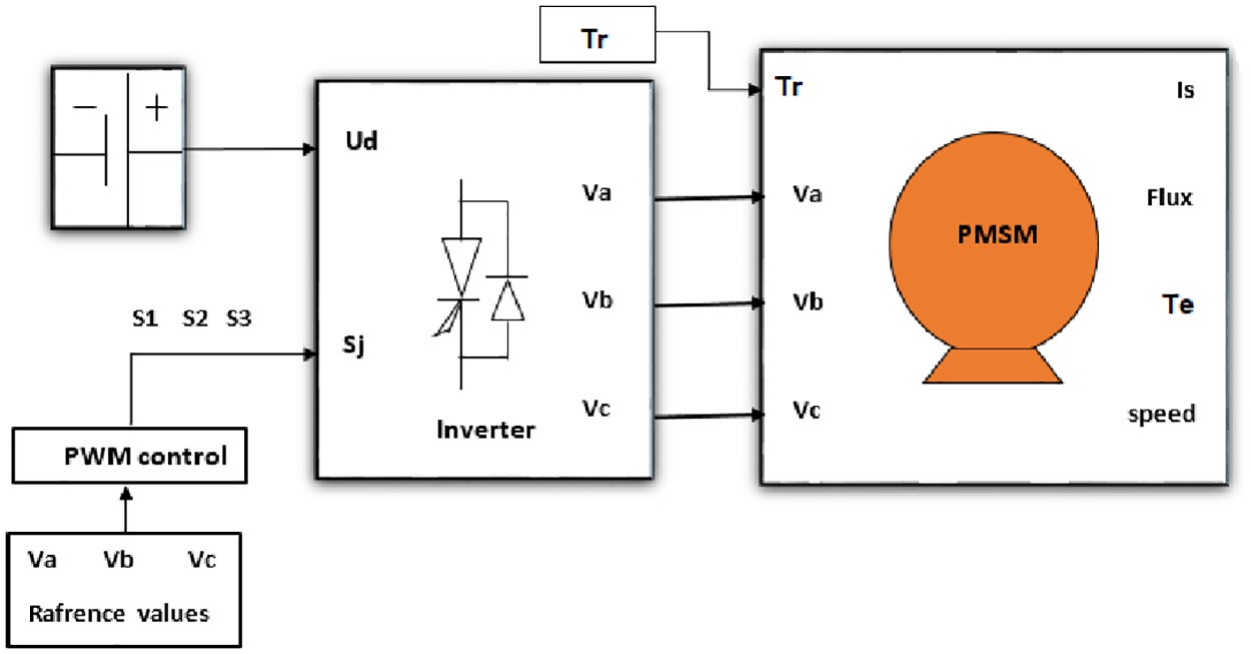
Schematic representation of a PMSM in open-loop.

The input of the motor is a three phase time dependent (AC) voltage source that regulates the phase currents to allow the control of the motor’s electromagnetic (ELM) torque or speed. The motor is fed from the grid to convert electrical energy into mechanical energy which is made up of the speed and torque of the motor. When controlling the motor, it must be fed to the voltage source inverter (VSI) with its control logic. The inverter’s role is to transform the DC voltage to AC voltage of variable magnitude and frequency. Fig 3 represents a general block diagram of a PMSM in open loop configuration [12, 24].

## Design of the vector control

**Fig 4:**
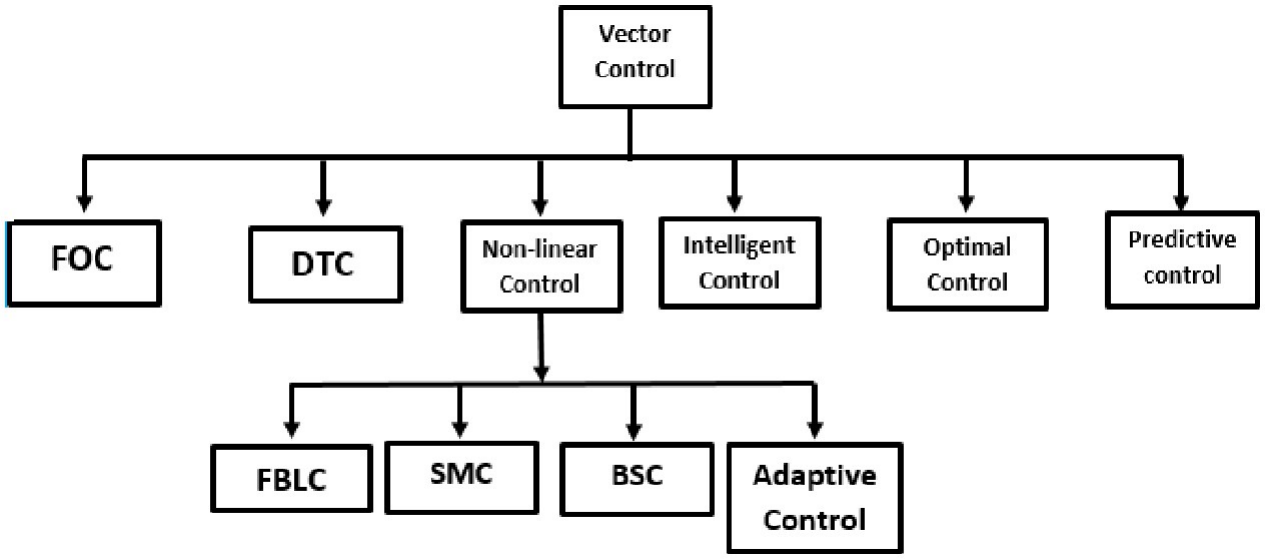
Classification of vector control [27].

The control design approaches to the PMSMs can be broadly classified into scalar and vector control. The first one is easy to implement and provide a relatively steady-state response but it pose the problem of slower dynamics. Therefore, in order to realize a higher accuracy and good dynamic and steady-steady response, closed loop vector control approach is usually preferred. The vector control is the largest control group, which includes the conventional, nonlinear, optimal, adaptive and intelligent techniques. In general, The vector control schemes of PMSM are Field-Oriented Control (FOC) and Direct Torque Control (DTC) as represented in Fig 4 [6, 25, 26].

### Design of field oriented control (FOC)

**Fig 5:**
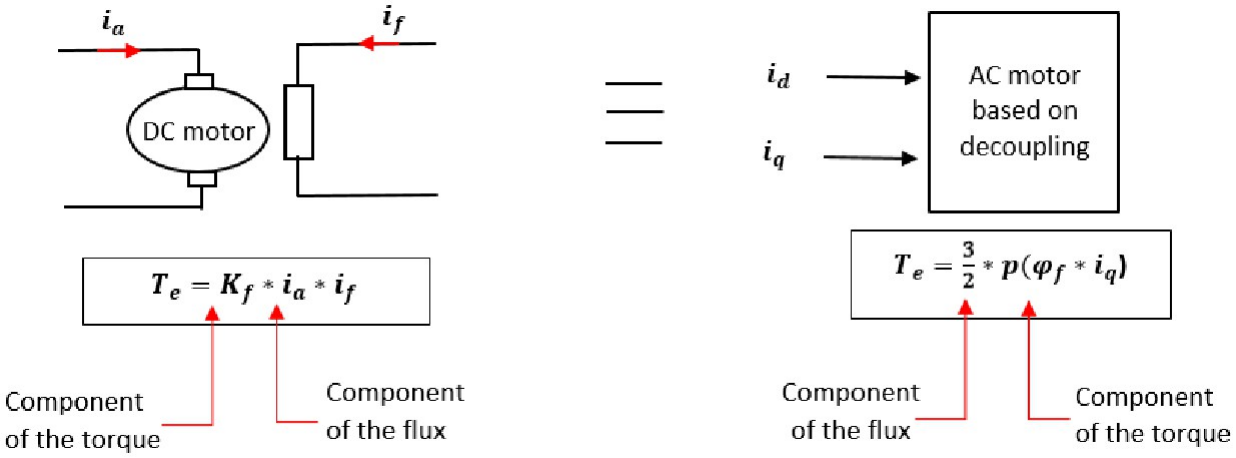
Equivalence between decoupled DC motor and PMSM.

The objective of a Field-Oriented Control applied to PMSM is to arrive at a model equivalent to that of a DC machine (decoupled linear model). One such model consists of maintaining the component *I*_*d*_ equal to zero as shown in Fig 5. Where *K*_*f*_ is a constant, *p* is the number of rotor permanent magnet pole pairs, *i*_*a*_ and *i*_*f*_ are respectively the induced and the inductor current of the DC motor respectively.

**Fig 6:**
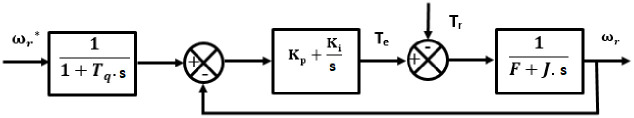
Closed loop speed regulation.

**Fig 7:**
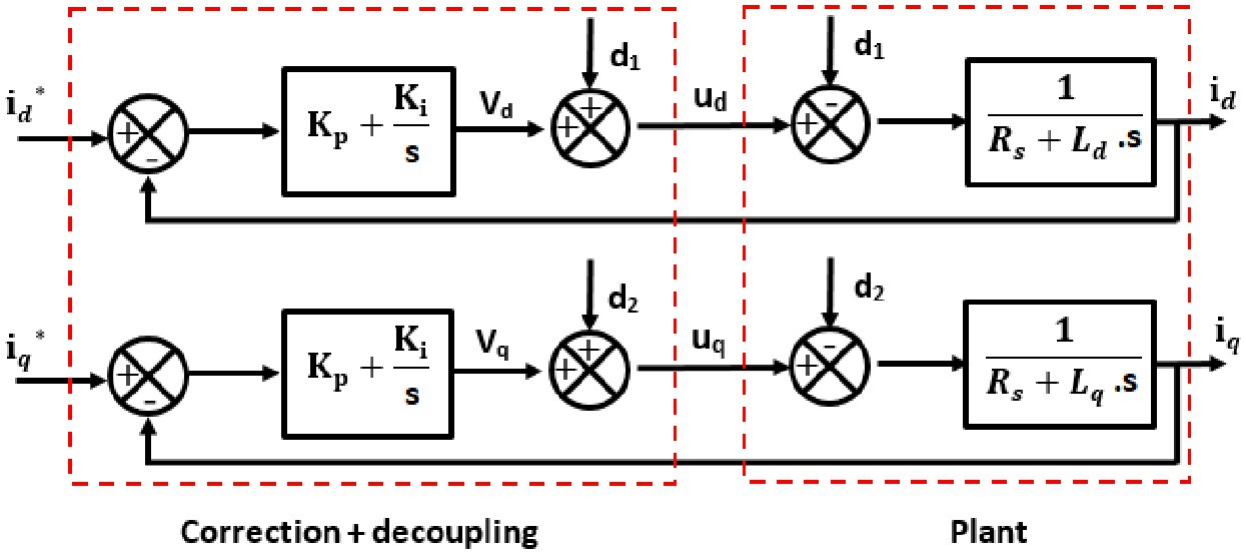
Closed loop regulation of current.

Here, the torque is controlled by making use of the current only. For this control, a speed loop and a current loop is used as shown in Figs 6 and 7 respectively.

**Fig 8:**
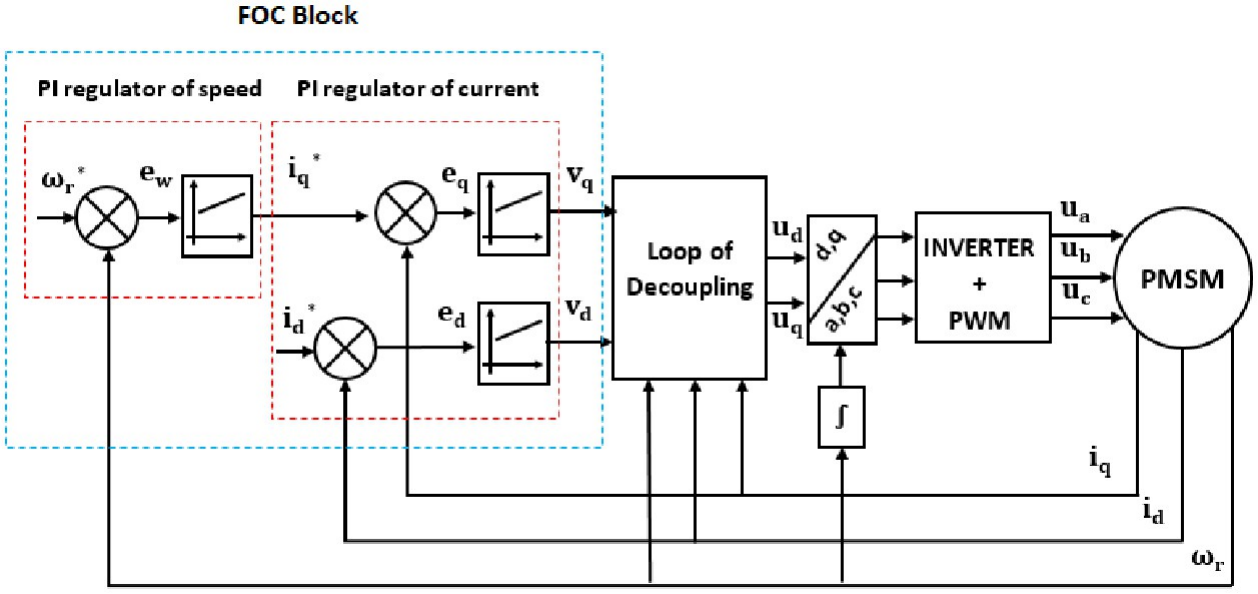
Generalized scheme for the Field Oriented Control (FOC) of PMSM.

A generalized FOC scheme is shown in Fig 8.

#### Speed control loop

After linearization of the torque expression in Eq (3), the simplified dynamics are as follows:

$$\begin{array}{c} T_{e}=\frac{3.p}{2}(\Phi_{f}.i_{q}) \end{array}$$ (3)

Moreover, a filter can be added in the speed control loop in order to minimize the overshoot as shown in Fig 6. The closed loop transfer function of this speed regulation loop is calculated as in Eq (4):

$$\begin{array}{c} TFCL\left(s\right)=\frac{\omega (s)}{\omega_{r}^{*}\left(s\right)}=\frac{1+\tau .s}{\frac{J}{K_{i}}.s^{2}+\tau .s+1} \end{array}$$ (4)

where *τ* is the time constant of the filter. As noticed, the characteristics of the transfer function is that of second order, so the proportional (*K*_*p*_) and integral (*K*_*i*_) gains are given in Eq (5):

$$\begin{array}{c} \left\{ \begin{array}{cc} K_{i}=\frac{4.J}{\tau^{2}} & \;\\ K_{p}=K_{i}.T_{q} & \; \end{array}\right. \end{array}$$ (5)

where: $\tau =\frac{2.\xi }{\omega_{0}}$ and $\tau =T_{q}=\frac{L_{q}}{R_{s}}$ and *ξ* is the damping coefficient and *ω*_0_ is the pulsating frequency in rad/s.

#### Current control loop

The closed-loop equation of the first order dynamic system based on the model of PMSM represented in Eq (1) can be developed from Eqs (6), (7) and (8) as under:

$$\begin{array}{c} \left\{ \begin{array}{c} u_{d}=v_{d}+d_{1}\;\\ u_{q}=v_{q}+d_{2}+d_{3} \end{array}\right. \end{array}$$ (6)

where:

$$\begin{array}{c} \left\{ \begin{array}{c} v_{d}=i_{d}.\left(R_{s}+L_{d}.s\right)\;\\ v_{q}=i_{q}.\left(R_{s}+L_{q}.s\right) \end{array}\right. \end{array}$$ (7)

and

$$\begin{array}{c} \left\{ \begin{array}{c} d_{1}=-L_{d}.\omega_{e}.i_{d}\;\\ d_{2}=L_{q}.\omega_{e}.i_{q}+\Phi_{f}.\omega_{e}\; \end{array}\right. \end{array}$$ (8)

The closed loop transfer function is given in Eq (9):

$$\begin{array}{c} TFCL\left(s\right)=\frac{1}{\frac{R_{s}}{K_{i}}.s+1} \end{array}$$ (9)

Also, it is straight forward to compute the constants of regulation for direct and quadratic currents as in Eqs (10) and (11).

$$\begin{array}{c} \left\{ \begin{array}{cc} K_{pd}=\frac{L_{d}}{T_{d}} & \;\\ K_{pq}=\frac{L_{q}}{T_{q}} & \; \end{array}\right. \end{array}$$ (10)

$$\begin{array}{c} \left\{ \begin{array}{cc} K_{id}=\frac{R_{s}}{T_{d}} & \;\\ K_{iq}=\frac{R_{s}}{T_{q}} & \; \end{array}\right. \end{array}$$ (11)

Where, $T_{d}=\frac{L_{d}}{R_{s}}$ and $T_{q}=\frac{L_{q}}{R_{s}}$

### Design of feedback linearization control (FBLC)

Feedback linearization is based on a change of variables and a suitable input to cancel the system’s nonlinearities which results in a closed-loop linear system. To achieve FBLC, the original system must be converted into an equivalent simpler model. Canceling the nonlinearities and imposing the desired linear dynamics can be applied if the PMSM system is as follows [17, 28]: The system dynamics are given by a model in Eq (12) with subsequent details in Eqs (13) and (14) as:

$$\begin{array}{c} \left\{ \begin{array}{cc} \dot{x}\left(t\right)=f\left(x\right)+g\left(x\right).u\left(t\right) & \;\\ y(t)=h(x) & \; \end{array}\right. \end{array}$$ (12)

where:

$$\begin{array}{c} \left\{ \begin{array}{cc} x\left(t\right)={\left[\begin{array}{ccc} i_{d} & i_{q} & \omega_{e} \end{array}\right]}^{T} & \;\\ u\left(t\right)={\left[\begin{array}{cc} u_{d} & u_{q} \end{array}\right]}^{T} & \;\\ y\left(t\right)=\omega_{r} \end{array}\right. \end{array}$$ (13)

and

$$\begin{array}{c} \left\{ \begin{array}{cc} f\left(x\right)={\left[\begin{array}{ccc} f_{1}\left(x\right) & f_{2}\left(x\right) & f_{3}\left(x\right) \end{array}\right]}^{T} & \;\\ g\left(x\right)=[\begin{array}{cc} \frac{1}{L_{d}} & 0\\ 0 & \frac{1}{L_{q}}\\ 0 & 0 \end{array}]\; \end{array}\right. \end{array}$$ (14)

where *f*_1_(*x*), *f*_2_(*x*) and *f*_3_(*x*) are computed from the transformation of the PMSM in the nonlinear method.

Since, the nonlinear model of the PMSM is explained in above equations, it is convenient to see that our control variables are the direct current and angular speed. It is straight forward to apply this control scheme step by step as follow:

#### For the first output *i*_*d*_

$$\begin{array}{c} \left\{ \begin{array}{cc} y_{1}=i_{d}=h_{1}\left(x\right) & \;\\ {\dot{y}}_{1}=L_{f}h_{1}\left(x\right)=-\frac{R_{s}}{L_{d}}.i_{d}+\\ \frac{L_{q}}{L_{d}}.p.\omega_{r}.i_{d}+\frac{1}{L_{d}}.u_{d} \end{array}\right. \end{array}$$ (15)

#### For the second output *ω*_*m*_

$$\begin{array}{c} \left\{ \begin{array}{cc} y_{2}=\omega_{r}=h_{2}\left(x\right) & \;\\ {\dot{y}}_{2}=\frac{3.p}{2.J}(\Phi_{f}.L_{q}+(L_{d}-L_{q}).i_{d}.i_{q})-\\ \frac{1}{J}.T_{r}-\frac{F}{J}.\omega_{r} & \;\\ {\ddot{y}}_{2}=K_{t}\left(L_{d}-L_{q}\right).i_{d}.f_{1}\left(x\right)+\\ K_{t}(\Phi_{f}+(L_{d}-L_{q})i_{d})f_{2}\left(x\right) & \;\\ -(\frac{1}{J}.T_{r}+\frac{F}{J})f_{3}\left(x\right)+\frac{K_{t}}{L_{q}}\left(L_{d}-L_{q}\right).i_{d}.u_{q}+\\ \frac{K_{t}}{L_{q}}(\Phi_{f}+(L_{d}-L_{q}).i_{d}).u_{d} & \; \end{array}\right. \end{array}$$ (16)

where: $K_{t}=\frac{3.p}{2.J}$ Since the relative degree is *r*_1_+ *r*_2_ = 3 = *n* (order the system), we have:

$$\begin{array}{cc} {\left[\begin{array}{c} {\dot{y}}_{1}{\ddot{y}}_{2} \end{array}\right]}^{T} & =a(x)+b(x).u\; \end{array}$$ (17)

Therefore, the nonlinear terms are cancelled out by choosing a transformation as:

$$\begin{array}{c} \left[\begin{array}{c} u_{d}\\ u_{q} \end{array}]=b^{-1}\left(x\right)([\begin{array}{c} v_{1}\\ v_{2} \end{array}]-a\left(x\right)\right) \end{array}$$ (18)

Where the b(x) matrix is smooth. After canceling the non-linearity of the PMSM dynamic system, the vector control is defined by the Eq (17):

$$\begin{array}{c} \left[\begin{array}{c} v_{1}\\ v_{2} \end{array}]=[\begin{array}{c} {\dot{e}}_{d}+K_{d}.e_{d}\\ {\ddot{e}}_{\omega }+K_{w1}.{\dot{e}}_{\omega }+K_{w2}.e_{\omega } \end{array}\right] \end{array}$$ (19)

where:

$$\begin{array}{c} \left\{ \begin{array}{cc} e_{1}={i^{*}}_{d}-i_{d} & \;\\ e_{\omega }={\omega^{*}}_{r}-\omega_{r} & \; \end{array}\right. \end{array}$$ (20)

**Fig 9:**
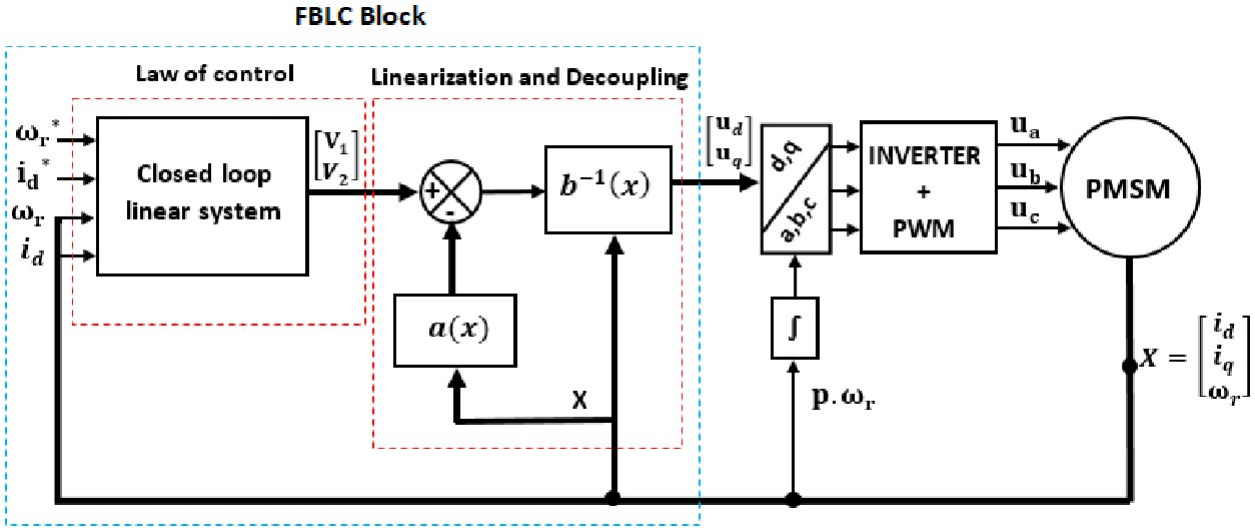
Block diagram of PMSM controlled by FBLC.

The simulation of the nonlinear control of the PMSM based on FBLC is shown in Fig 9.

### Design of backstepping control (BSC)

Backstepping control is another popular nonlinear control technique to study the stabilization of the system by making use of the Lyapunov function candidate [29]. It has multi-steps, every step of the process generates a virtual control to ensure the convergence of the system towards its state of equilibrium. The model of PMSM is based on the state-space model given in Eq (21) where the variable states are direct and quadratic current and mechanical speed [8, 30]:

$$\begin{array}{c} \left\{ \begin{array}{cc} \frac{di_{d}}{dt}=\frac{1}{L_{d}}.\left(u_{d}-R_{s}.i_{d}+\omega_{e}.L_{q}.i_{q}\right) & \;\\ \frac{di_{q}}{dt}=\frac{1}{L_{q}}.\left(u_{q}-R_{s}.i_{q}-\omega_{e}.L_{d}.i_{d}-\omega_{e}.\Phi_{f}\right) & \;\\ \frac{d\omega_{r}}{dt}=\frac{1}{J}.\left(T_{e}-T_{r}-F.\omega_{r}\right) & \; \end{array}\right. \end{array}$$ (21)

where:

$$\begin{array}{cc} T_{e}=\frac{3}{2}.p(\left(L_{d}-L_{q}\right).i_{d}.i_{q}+\Phi_{f}.i_{q}) & \; \end{array}$$ (22)

The dynamic errors are given as follows:

$$\begin{array}{c} \left\{ \begin{array}{cc} e_{\omega }=\omega_{r}^{*}-\omega_{r} & \;\\ e_{d}=i_{d}^{*}-i_{d} & \;\\ e_{q}=i_{q}^{*}-i_{q} & \; \end{array}\right. \end{array}$$ (23)

The backstepping control applied on PMSM requires the understanding of two major steps as follows:

#### Step-1

In the first step, we study electromechanical decoupling to use the candidate Lyapunov function (CLF) to ensure the same reference by ensuring the orientation of direct current $i_{d}^{*}=0$ to optimize the copper loss. The CLF and its derivative is defined as:

$$\left\{ \begin{array}{cc} V_{1}=\frac{1}{2}.{e^{2}}_{\omega } & \left(V_{1}>0\right)\\ {\dot{V}}_{1}=e_{\omega }.{\dot{e}}_{\omega } & \left({\dot{V}}_{1}<0\right) \end{array}\right.$$

This choice will result in zero error and the system will be stable. Therefore:

$$\begin{array}{c} \left\{ \begin{array}{cc} {\dot{e}}_{\omega }=-K_{\omega }.e_{\omega } & \;\\ {\dot{V}}_{1}=-K_{\omega }.{e^{2}}_{\omega } & \; \end{array}\right. \end{array}$$ (24)

In the closed-loop, the tracking error of the angular speed is presented in Eq (25):

$$\begin{array}{cc} {\dot{e}}_{\omega }={\dot{\omega }}_{r}^{*}-\frac{3.p}{2.J}\left(\Phi_{f}.i_{q}\right)+\frac{F}{J}.\omega_{e}+\frac{1}{J}.T_{r} & \; \end{array}$$ (25)

As the reference is a step function, the reference of the direct current is given as follows:

$$\begin{array}{cc} i_{q}^{*}=\frac{2}{3.p^{2}.\Phi_{f}}\left(J.K_{\omega }.e_{\omega }+F.+\omega_{r}+p.T_{r}\right) & \; \end{array}$$ (26)

#### Step-2

In the second step, we put direct and quadratic current for the virtual control and after that, we conclude the real control voltage as below:

$$\left\{ \begin{array}{cc} V_{2}=\frac{1}{2}.{e^{2}}_{\omega }+\frac{1}{2}.{e^{2}}_{d}+\frac{1}{2}.{e^{2}}_{q} & \left(V_{2}>0\right)\\ {\dot{V}}_{2}=e_{\omega }.{\dot{e}}_{\omega }+e_{d}.{\dot{e}}_{d}+e_{q}.{\dot{e}}_{q} & \left({\dot{V}}_{2}<0\right) \end{array}\right.$$

For the stable system, we can write as:

$$\begin{array}{c} \left\{ \begin{array}{c} {\dot{e}}_{d}=-K_{d}.e_{d}=\frac{R_{s}}{L_{d}}.i_{d}-\frac{L_{q}}{L_{d}}.p.\omega_{r}.i_{q}-\frac{1}{L_{d}}.u_{d}\;\\ {\dot{e}}_{q}=-K_{q}.e_{q}=\left(\frac{2}{3.p^{2}.\Phi_{f}}\right)\left(F-K_{\omega }\right)(\frac{3.p^{2}.\Phi_{f}}{2}i_{q}-\;\\ F.\omega_{r}-p.T_{r})+\frac{R_{s}}{L_{q}}.i_{q}-\frac{L_{d}}{L_{q}}.\omega_{r}.i_{q}+\frac{\Phi_{f}}{i_{d}}.\omega_{r}-\\ \frac{1}{L_{q}}.u_{q}\; \end{array}\right. \end{array}$$ (27)

Finally, the control law was obtained as follows:

$$\begin{array}{c} \left\{ \begin{array}{c} u_{d}=-L_{d}\left(-\frac{R_{s}}{L_{d}}.i_{d}+\frac{L_{q}}{L_{d}}.p.\omega_{r}.i_{q}-K_{d}.e_{d}\right)\;\\ u_{q}=-L_{q}(-\left(\frac{2}{3.p^{2}.\Phi_{f}}\right)\left(F-K_{\omega }\right)(\frac{3.p^{2}.\Phi_{f}}{2}i_{q}-\;\\ F.\omega_{r}-p.T_{r})-\frac{R_{s}}{L_{q}}.i_{q}-\frac{L_{d}}{L_{q}}.\omega_{r}.i_{q}-\frac{\Phi_{f}}{i_{d}}.\omega_{r}-\\ K_{q}.e_{q})\; \end{array}\right. \end{array}$$ (28)

**Fig 10:**
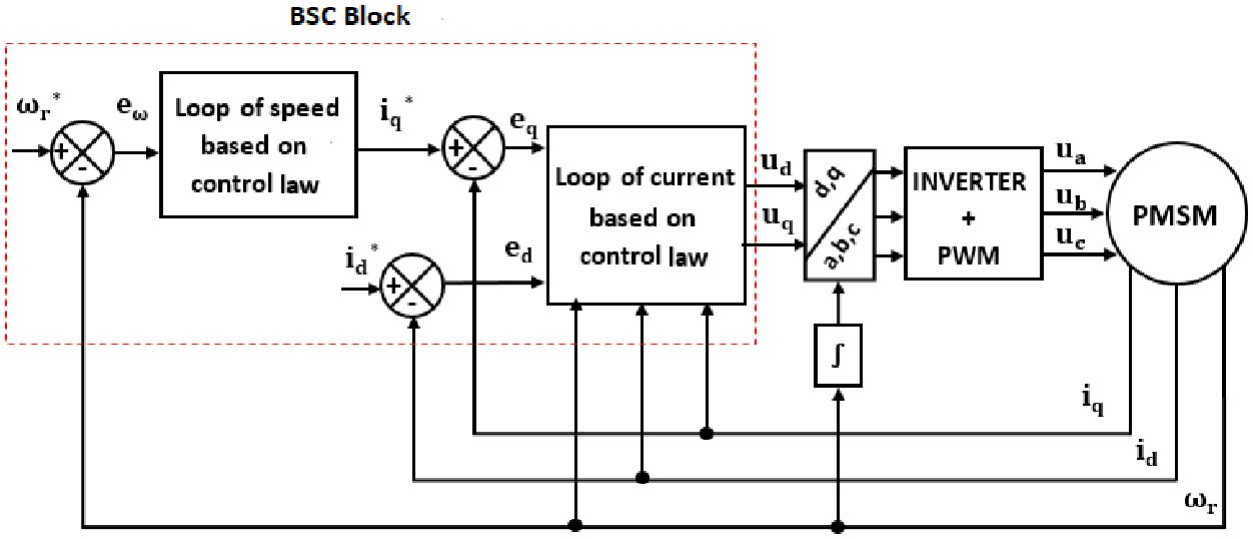
Block diagram of BSC control applied to PMSM.

The simulation of the control architecture of PMSM based on BSC is given in Fig 10:

### Design of sliding mode control (SMC)

The sliding mode control is one of the most popular nonlinear control as it offers high robustness under uncertainty and external disturbances. Luckily, it is characterized by a simple structure and fast tracking response against a reference input. Despite the interesting features of the classical SMC (C-SMC) mentioned above, the two main problems are the chattering phenomenon and a finite-time convergence, which appears as an oscillation and occurs in a steady state that may excite unmodelled high-frequency dynamics in the system [31]. In order to overcome this problem and give better performance characteristics, this paper presents the continuous approximation method based on SMC (CAM-SMC) applied on the current and speed loop of the PMSM drive [13, 15, 16]. Its design require two assignments. The first step is to calculate the characteristics of the controlled system with the desired dynamics through identified switching hyperplane. The second one is dependent on the discontinuous control in order to enter the system into the sliding mode *s*(*x*, *t*) = 0 and force it to stay there. Furthermore, the sliding surface design is the initial step on which SMC depends for the computation of control law [32, 33]. The general statement for sliding surface is defined in Eq 29 [34, 35].

$$\begin{array}{c} S\left(x,t\right)={\left(\frac{d}{dt}+\lambda \right)}^{n-1}.e\left(t\right) \end{array}$$ (29)

Where λ is a positive number chosen by the designer (scaling factor), n is system order, *S*(*x*, *t*) is the sliding surface and *e*(*t*) is tracking error. After designing the sliding surface, the system trajectory will be forced and attracted across it even to the origin reach asymptotically using the sliding condition shown in Eq 30 [36]:

$$\begin{array}{c} \frac{1}{2}.\frac{d}{dt}S^{2}\leq \eta \left|S\right| \end{array}$$ (30)

Where *η* > 0. Using the sliding surface and sliding condition, the control law is calculated using two phases. The first one is sliding phase in order to keep the system on the sliding surface by introducing an equivalent term, i.e. *S*(*x*, *t*) = 0 and $\dot{S}\left(x,t\right)=0$. The second one is an approaching phase in order to satisfy the sliding condition by designing the switching law for *S*(*x*, *t*)≠0 and *S*(*x*, *t*) = 0. The control law of SMC is calculated as follows:

$$\begin{array}{c} u=u_{eq}+u_{s} \end{array}$$ (31)

**Fig 11:**
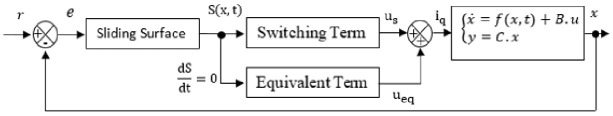
Block diagram of SMC based nonlinear plant.

The scheme of SMC based nonlinear system is shown in Fig 11. The proposed SMC design for PMSM model has two loops: one for speed and the other for current. Based on these loops, the SMC control is applied for SISO plant where the first order sliding surface is designed as follows:

$$\begin{array}{c} \left\{ \begin{array}{cc} S_{\omega }=\omega_{r}^{*}-\omega_{r} & \;\\ S_{d}=i_{d}^{*}-i_{d} & \;\\ S_{q}=i_{q}^{*}-i_{q} & \; \end{array}\right. \end{array}$$ (32)

Depending on the sliding phase, the equivalent terms of both PMSM’s loops are written as:

$$\begin{array}{c} \left\{ \begin{array}{c} i_{q-eq}^{*}=\frac{2.J}{3.p^{2}.\Phi_{f}}.\left({\dot{\omega }}_{r}^{*}+\frac{F}{J}.\omega_{r}+\frac{p}{J}.T_{l}\right)\;\\ u_{d-eq}=R_{s}.i_{d}-L_{q}i_{q}.\omega_{r}\;\\ u_{q-eq}=i_{q-eq}^{*}+R_{s}.i_{q}+L_{d}i_{d}.\omega_{r}+\Phi_{f}.\omega_{r}\; \end{array}\right. \end{array}$$ (33)

Also depending on the approaching phase, the switching terms of both speed and current loops of PMSM can be formulated as:

$$\begin{array}{c} \left\{ \begin{array}{c} i_{q-s}^{*}=K_{\omega }.tanh\left(S_{\omega }\right)\;\\ u_{d-s}=K_{d}.tanh(S_{d)}\\ u_{q-s}=K_{q}.tanh(S_{q)\;} \end{array}\right. \end{array}$$ (34)

Finally, the control laws based on SMC has been designed as follows:

$$\{\begin{array}{l} i_{q}^{*}=i_{q-eq}^{*}+i_{q-s}^{*}\\ u_{d}=u_{d-eq}+u_{d-s}\\ u_{q}=u_{q-eq}+u_{q-s} \end{array}$$ (35)

**Fig 12:**
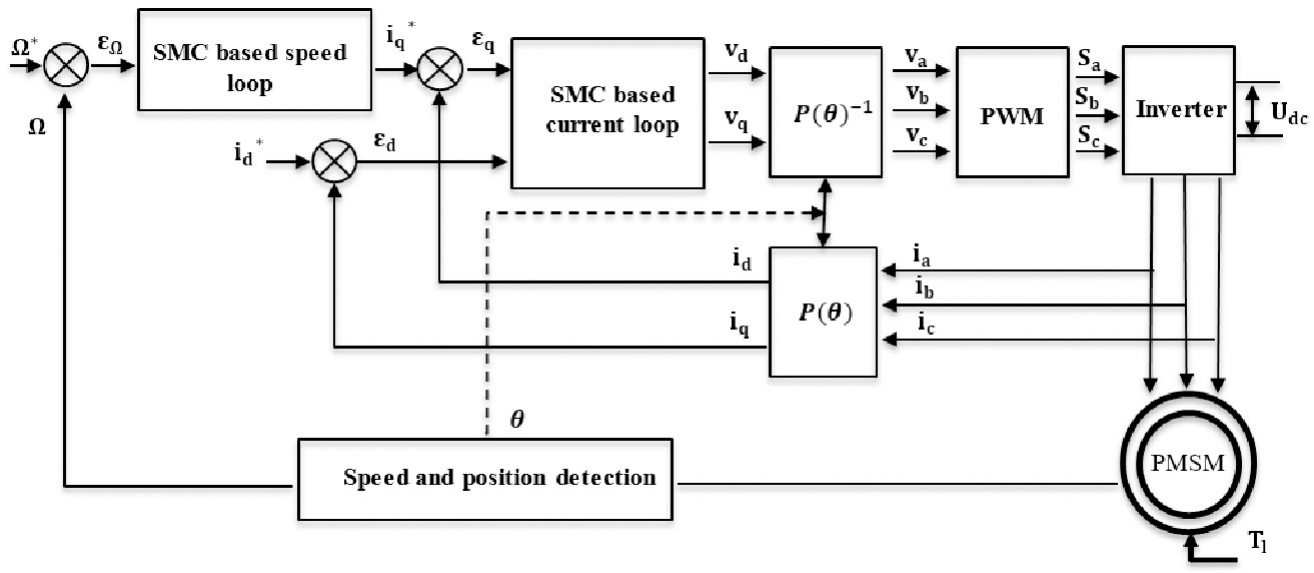
Block diagram of SMC control applied to PMSM.

The simulation of the control architecture of PMSM based on SMC is given in Fig 12:

## Simulation results & discussion

In this section, Matlab/Simulink is used for the simulation of FOC, FBLC, BSC and SMC respectively as applied for the control of a PMSM in flight control actuation of a MEA. The PMSM parameters that are used in the simulation are shown in Table 1. Among them, SMC parameters designed in this paper are *K*_*ω*_ = 20, *K*_*d*_ = 25, *K*_*q*_ = 25, BSC parameters are *K*_*ω*_ = 3 × 10^4^, *K*_*d*_ = 1 × 10^4^, *K*_*q*_ = 9 × 10^3^, FBLC parameters are *K*_*ω*1_ = 44 × 10^3^, *K*_*ω*2_ = 55 × 10^3^, *K*_*d*_ = 6 × 10^6^ and the FOC parameters have been given in Eqs (5), (10) and (11). To analyze and compare the best controller among these four; two aspects of the PMSM must be studied which are performance characteristics and robustness of the control technique to be used for MEA actuator design. For this purpose, six scenarios are used as follows:

**Table 1:** Simulation parameters of PMSM.

PMSM’s parameters			
*Description*	*Symbol*	*Value*	*Unit*
Stator Resistance per phase	*R*_*s*_	0.6	Ω
Stator d-axis Inductance	*L*_*d*_	1.4 × 10^−3^	H
Stator q-axis Inductance	*L*_*q*_	2.8 × 10^−3^	H
Rotor Inertia	*J*	1.1 × 10^−3^	kg.m^2^
Friction constant of the rotor	*F*	1.4 × 10^−3^	N.m.s^-1^
Rotor magnetic flux linking the stator	Φ_*f*_	12 × 10^−2^	Wb
Input voltage	*v*_*dc*_	100	V
Number of pole pairs	*p*	4	-

### Rotor speed tracking a fixed reference without a load torque

**Fig 13:**
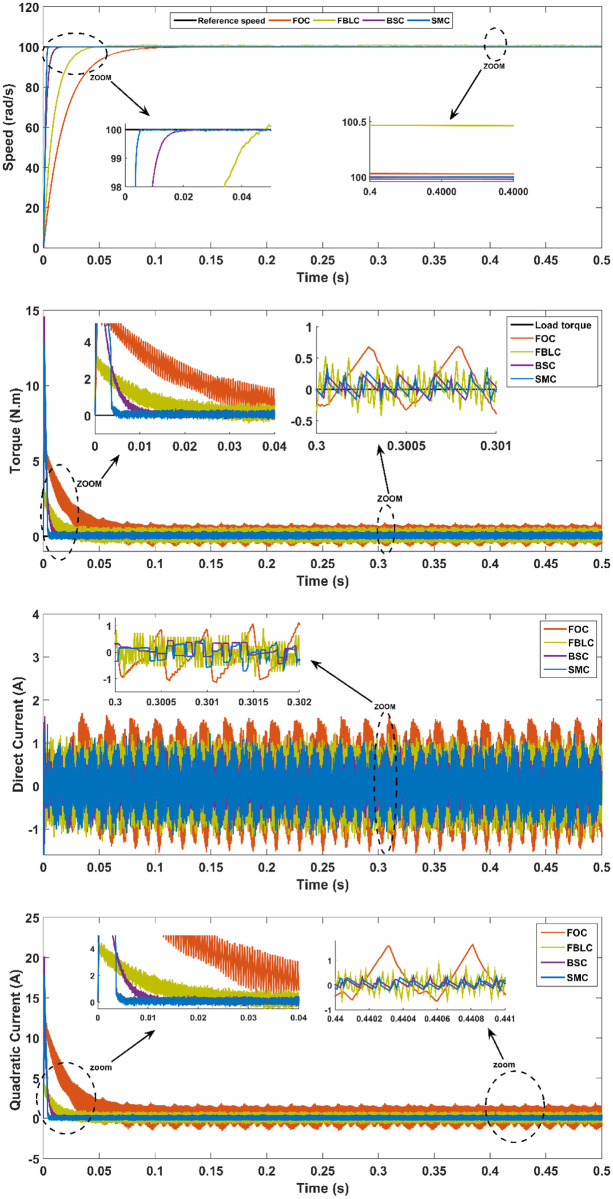
Scenario (1): Fixed reference tracking—No load.

In the first scenario, the value of rotor speed reference is set to 100 rad/s without applying a load torque as seen in Fig 13. From these results, the best performance is seen to be exhibited by the SMC followed by the FBLC and BSC; while, FOC shows the worst performance among the four controllers in terms of the steady-state error (*SSE*) and the settling time (*T*_*s*_).

### Rotor speed tracking a fixed reference with a load torque

**Fig 14:**
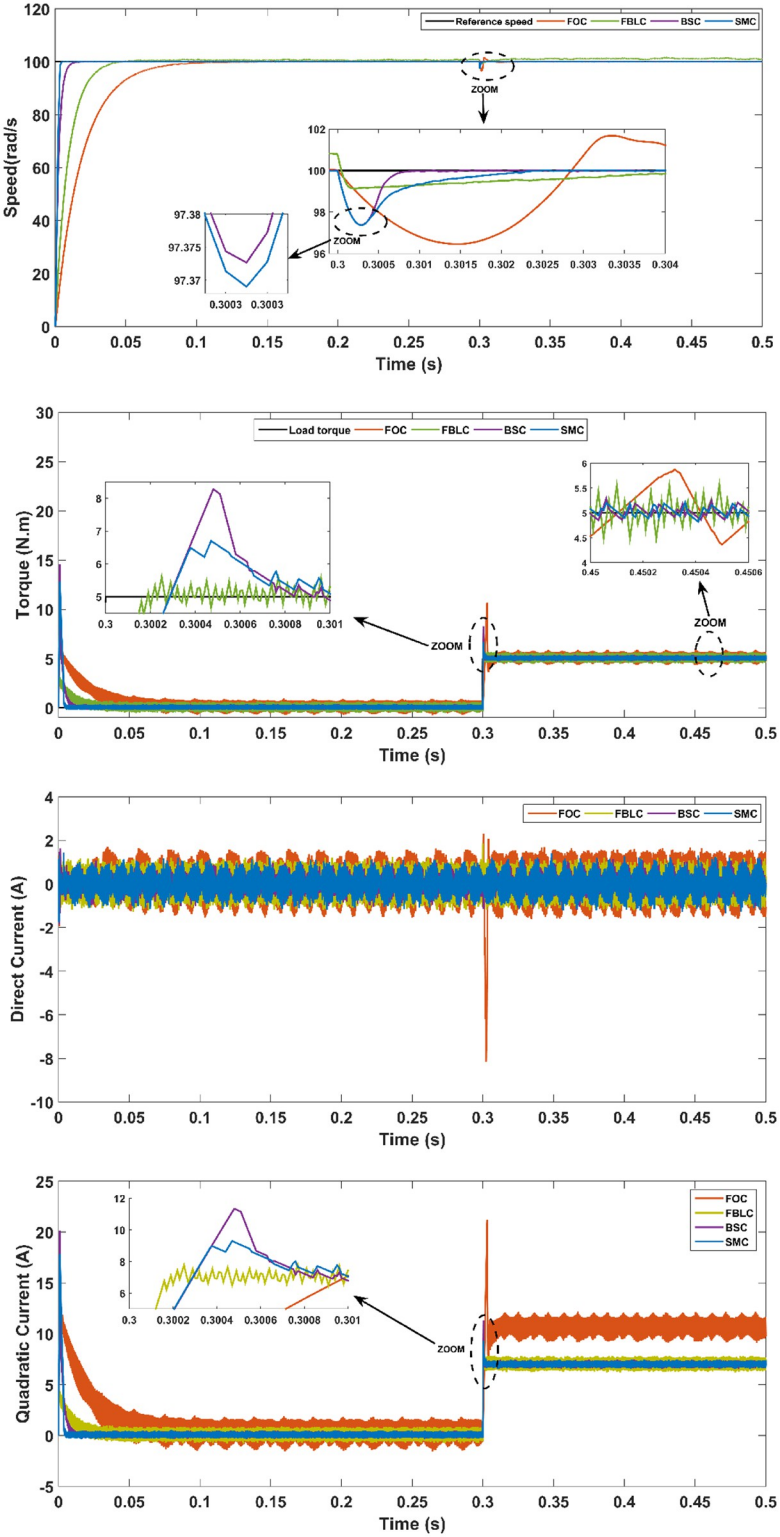
Scenario (2): Fixed reference tracking—With 5 N.m load applied at t = 0.3 s.

In this scenario, the reference value of the rotor speed tracking is a fixed value and a load torque of 5 N.m is applied at 0.3 s. After the application of the load torque, the performance has some undershoot and overshoot in which FBLC shows higher undershoot than either of the SMC, the BSC and FOC controllers. Moreover, SMC remained at the top in terms of other characteristics of PMSM’s performance like the overshoot during torque change, response time and steady-state error. The results of this scenario are represented in Fig 14.

### Rotor speed tracking a varying reference without a load torque

**Fig 15:**
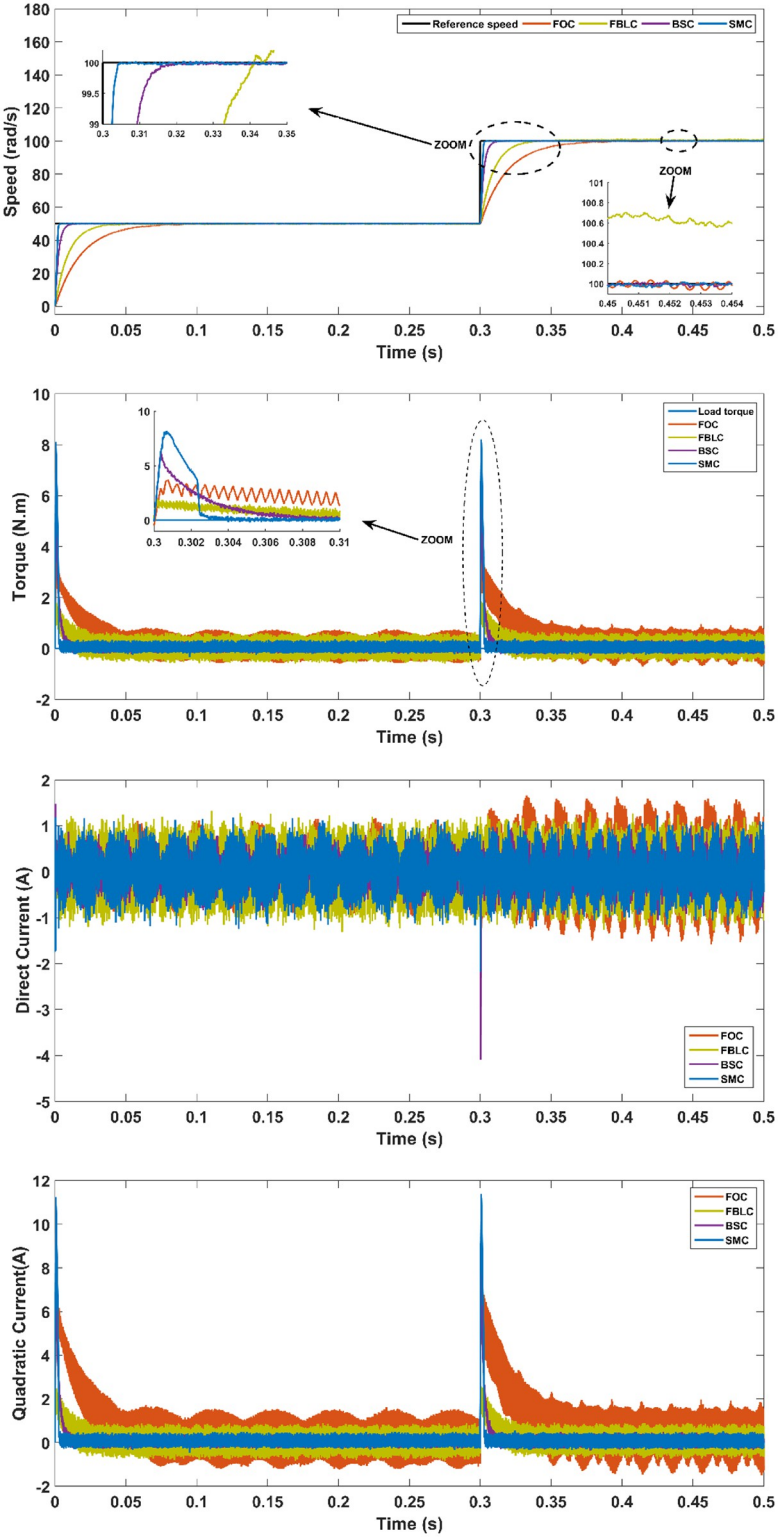
Scenario (3): Varying reference tracking—No load.

In this scenario, the reference value of the rotor speed is varied from 50 rad/s to 100 rad/s at 0.3 s without applying a load torque. This change leads to result in a peak overshoot in the ELM torque and quadratic current and therefore results in an increased steady-state error for direct current. However, the FBLC has lesser peak overshoot compared to the SMC, BSC and FOC respectively. Whereas, the SMC remained the best candidate in terms of all other characteristics of PMSM’s performance. Fig 15 clearly shows these results.

### Reference speed inversion with a load torque

**Fig 16:**
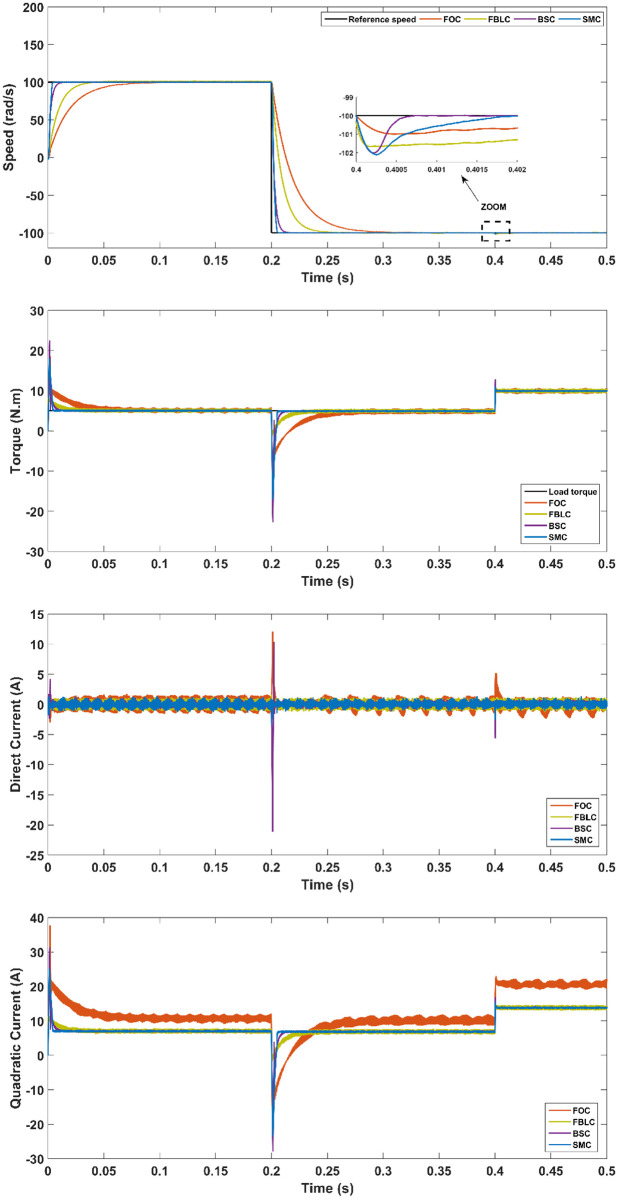
Scenario (4): Reference reversal tracking—With 10 N.m load applied at t = 0.4s.

To study the robustness of the vector control, a reversal reference value for the rotor speed reference (from +100 rad/s to -100 rad/s) is used and a load torque of 5 N.m is applied at t = 0.2 s. The results are represented in Fig 16 which shows better performance characteristics of the SMC as compared to BSC, FBLC and FOC. Whereas, the FBLC seems to be the best controller when applying the load torque as compared to SMC, BSC and FOC, especially for controlling the overshoot of the direct current.

### PMSM parameter variation with a load torque

**Fig 17:**
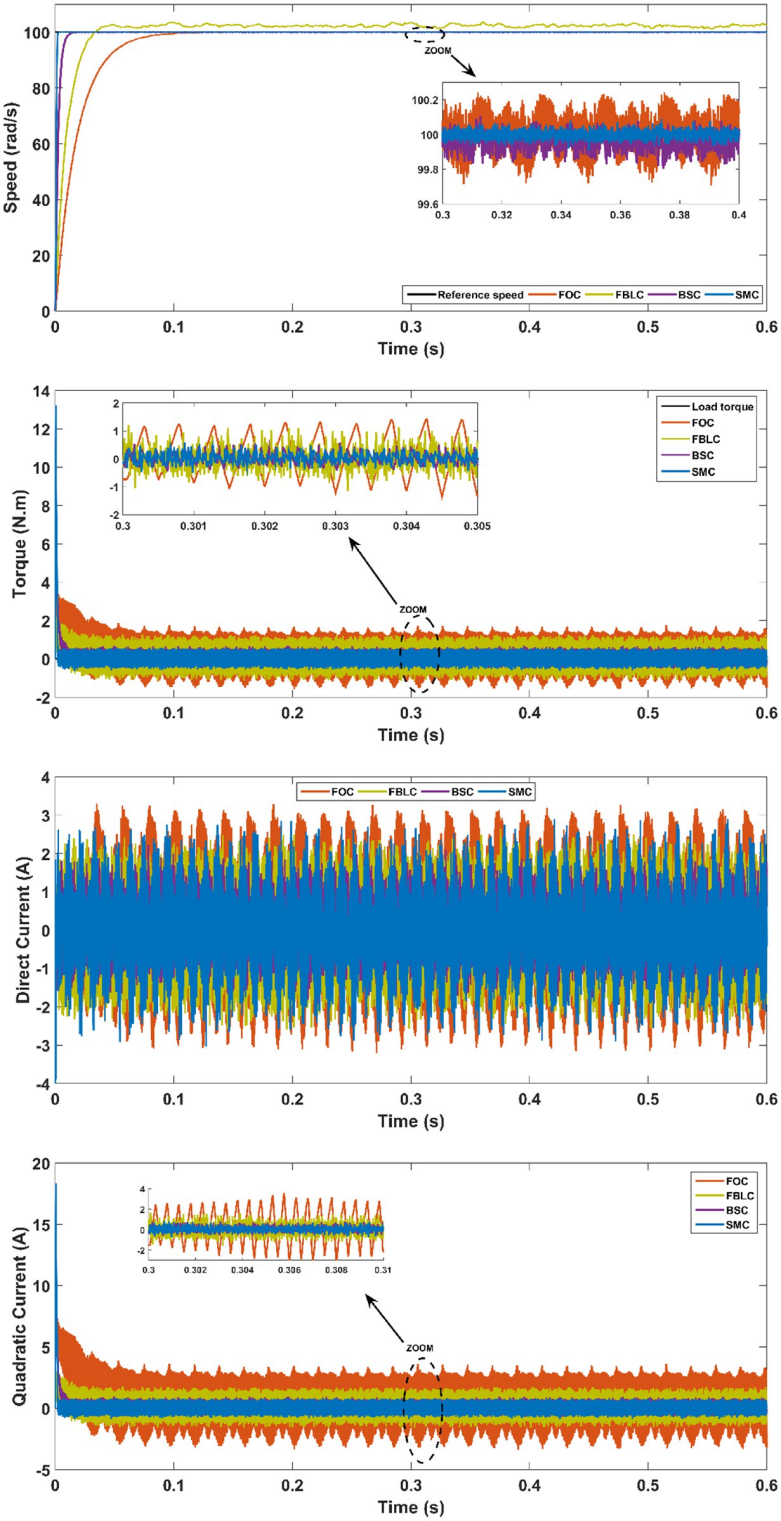
Scenario (5): Parameter variation (50% increase)—With 5 N.m load applied at t = 0.3s.

**Fig 18:**
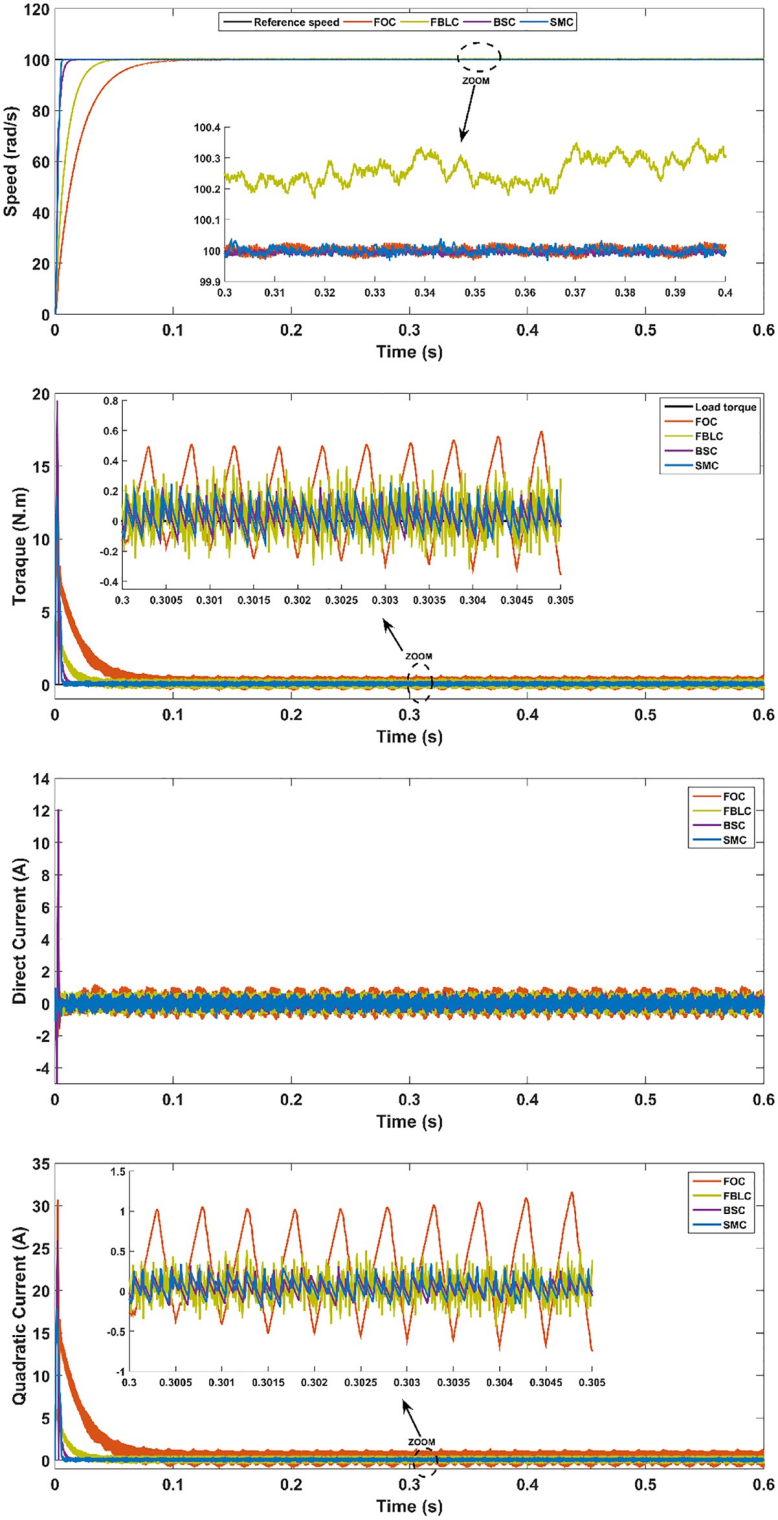
Scenario (6): Parameter variation (50% decrease)—With 5 N.m load applied at t = 0.3s.

In order to test the robustness under the parameter uncertainties of the PMSM, parameter values are decreased by 50% and increased by 50% of their nominal values in the fifth and sixth scenario respectively. This change results in impacting the steady-state error of the FBLC and FOC. It can be observed that although BSC performs well against uncertainties, however, SMC remains exceptional in terms of performance characteristics of the PMSM and robustness. The result of this scenario is shown in Figs 17 and 18 respectively.

Finally, in order to scrutinize the performance characteristics and durability of each controller accurately, the characteristics of PMSM’s performance for all six scenarios is summarized in Table 2 as under.

**Table 2:** Performance comparison of three PMSM vector controllers.

Controller Type	Performance Characteristics				
	Response time (ms)	Influence of Uncertainties	Performance Stability	Steady state error (%)	Tolerance band peak (%)
*FOC*	100	High	Good	0.05	18
*FBLC*	45	Medium	Quite Good	0.4	10
*BSC*	14	Low	Exceptional	0.03	5
*SMC*	4.5	Lowest	Exceptional	0.01	4

From the results, it is evident that under no-load conditions, the rise time for the SMC controller is only 0.005 s while BSC offers 0.01 sec, 0.025 s for FBLC and 0.05 s for FOC controller. Thus, SMC shows the fastest speed of response with close to zero steady state error as shown in Fig 13. Under load torque of 5 N.m, the SMC and BSC shows a decrease in speed up to 2.5 rad/s, while FBLC shows a speed reduction of only 1 rad/s while FOC results in largest change of 4 rad/s as shown in Fig 14. Also, large spikes in current are obtained for FOC response in the presence of disturbance torque. For the varying reference tracking in Fig 15, SMC response is fastest as in Fig 13 with some spikes in torque (and current) curve which quickly settles to zero in 0.002 s well ahead of the other three controllers. In the reference reversal scenario with 10 N.m load torque in Fig 16, BSC performs the best as it reaches the reference speed within 0.7 ms. SMC takes 1.7 s to settle although both controllers show the same reduction in speed as that of 2 rad/s. the strength of SMC is evident from Fig 17 where 50% increase in parameter values with 5 N.m load torque is simulated. All three controllers show significant oscillations where FOC exhibits ± 1 N.m peak to peak torque and ± 3A current variation. Whereas, 50% decrease in parameter values shows less significant effect in the PMSM response. FOC shows notable peak to peak variation of 0.7 N.m in torque and current values. SMC shows stable behaviour with ± 0.2 N.m peak to peak torque and current variation under these conditions as shown in Fig 18.

## Conclusion

More electric aircraft (MEA) are the way forward to realize all electric aircraft (AEA) technology where hydraulic and pneumatic systems will be completely replaced by electric actuators. In this paper, a comparative analysis of four vector control techniques i.e. Field-Oriented Control (FOC), Feedback Linearization (FBL), Backstepping (BSC) and continuous approximation based Sliding Mode Control (SMC) are presented for the PMSM based actuator design for flight control applications. The PMSM is modeled by using the generalized dynamical equations and the four control strategies are analyzed for the robust performance. Finally, the simulation results with six major scenarios are used to narrow down the controller which is able to withstand against uncertainties and disturbances. In this quest, although the Backstepping control performs well against uncertainties, however, in terms of overall performance including response time, steady state error and robustness, SMC outperforms all other control schemes. In future, sensorless control of motor drive with different nonlinear observers will be analyzed for performance comparison.
